# Bis(tetra­phenyl­phospho­nium) tetra­chlorido­cobaltate(II)

**DOI:** 10.1107/S1600536814011210

**Published:** 2014-05-21

**Authors:** Zeghouan Ouahida, Nasreddine Hadjadj, Fatiha Guenifa, Lamia Bendjeddou, Hocine Merazig

**Affiliations:** a Unité de Recherche Chimie de l’Environnement et Moléculaire Structurale (CHEMS), Faculté des Sciences Exactes, Campus Chaabet Ersas, Université Constantine I, 25000 Constantine, Algeria

## Abstract

The title compound, (C_24_H_20_P)_2_[CoCl_4_], was prepared under hydro­thermal conditions. In the crystal, the tetra­phenyl­phospho­nium cations are linked by pairs of weak C—H⋯π inter­actions into supra­molecular dimers; the Co^II^ cations lie on twofold rotation axes and the tetra­hedral [CoCl_4_]^2−^ anions are linked with the tetra­phenyl­phospho­nium cations *via* weak C—H⋯Cl hydrogen bonds.

## Related literature   

For background and applications of compounds with supramolecular structures, see: Rowsell & Yaghi (2005 ▶); Dong *et al.* (2007 ▶); Wu & Lin (2007 ▶); Zhao *et al.* (2003 ▶); Neville *et al.* (2008 ▶); Huang *et al.* (2007 ▶). For applications of the tetra­phenyl­phospho­nium ion in supra­molecular chemistry and numerous coordination polymers, see: Zacharie *et al.* (1985 ▶); Schlueter & Geiser (2007 ▶).
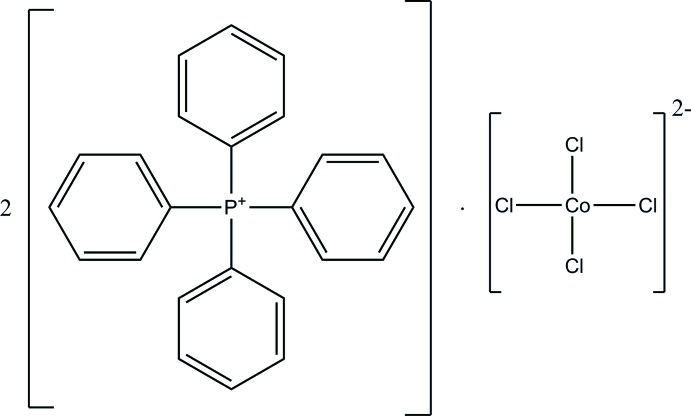


## Experimental   

### 

#### Crystal data  

(C_24_H_20_P)_2_[CoCl_4_]
*M* *_r_* = 879.46
Monoclinic, 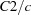

*a* = 10.9154 (4) Å
*b* = 19.2514 (6) Å
*c* = 20.1826 (7) Å
β = 91.008 (2)°
*V* = 4240.4 (3) Å^3^
*Z* = 4
Mo *K*α radiation
μ = 0.77 mm^−1^
*T* = 293 K
0.20 × 0.10 × 0.08 mm

#### Data collection  

Bruker APEXII diffractometer
12226 measured reflections
3748 independent reflections
3180 reflections with *I* > 2σ(*I*)
*R*_int_ = 0.028

#### Refinement  

*R*[*F* ^2^ > 2σ(*F* ^2^)] = 0.030
*wR*(*F* ^2^) = 0.084
*S* = 1.04
3748 reflections
249 parameters
H-atom parameters constrained
Δρ_max_ = 1.51 e Å^−3^
Δρ_min_ = −0.22 e Å^−3^

### 

Data collection: *APEX2* (Bruker, 2006 ▶); cell refinement: *SAINT* (Bruker, 2006 ▶); data reduction: *SAINT*; program(s) used to solve structure: *SIR2002* (Burla *et al.*, 2003 ▶); program(s) used to refine structure: *SHELXL97* (Sheldrick, 2008 ▶); molecular graphics: *ORTEP-3 for Windows* (Farrugia, 2012 ▶); software used to prepare material for publication: *WinGX* (Farrugia, 2012 ▶), *Mercury* (Macrae *et al.*, 2006 ▶) and *POVRay* (Persistence of Vision Team, 2004 ▶).

## Supplementary Material

Crystal structure: contains datablock(s) global, I. DOI: 10.1107/S1600536814011210/xu5791sup1.cif

Structure factors: contains datablock(s) I. DOI: 10.1107/S1600536814011210/xu5791Isup2.hkl

CCDC reference: 1003287

Additional supporting information:  crystallographic information; 3D view; checkCIF report

## Figures and Tables

**Table 1:** Selected bond lengths (Å)

Co1—Cl2	2.2791 (6)
Co1—Cl1	2.2873 (6)

**Table 2:** Hydrogen-bond geometry (Å, °) *Cg*2 and *Cg*4 are the centroids of the C19–C24 and C7-C12 benzene rings, respectively.

*D*—H⋯*A*	*D*—H	H⋯*A*	*D*⋯*A*	*D*—H⋯*A*
C3—H3⋯Cl1	0.93	2.80	3.552 (3)	138
C11—H11⋯Cl1^i^	0.93	2.81	3.633 (2)	148
C23—H23⋯Cl2^ii^	0.93	2.77	3.644 (2)	156
C14—H14⋯*Cg*4^iii^	0.93	2.88	3.650 (2)	141
C21—H21⋯*Cg*2^iii^	0.93	2.79	3.446 (2)	129

Symmetry codes: (i) 
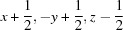
; (ii) 
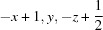
; (iii) 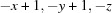.

**Fig. 1.**
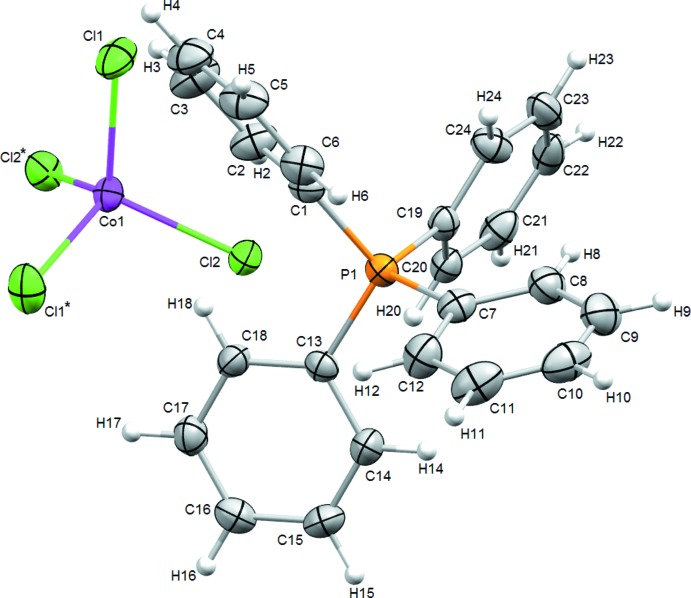
The asymmetric unit of the title structure with the atom numbering scheme. Displacement ellipsoids are drawn at the 50% probability level. [Symmetry code: (*): -x, y, 1/2 - z].

**Fig. 2.**
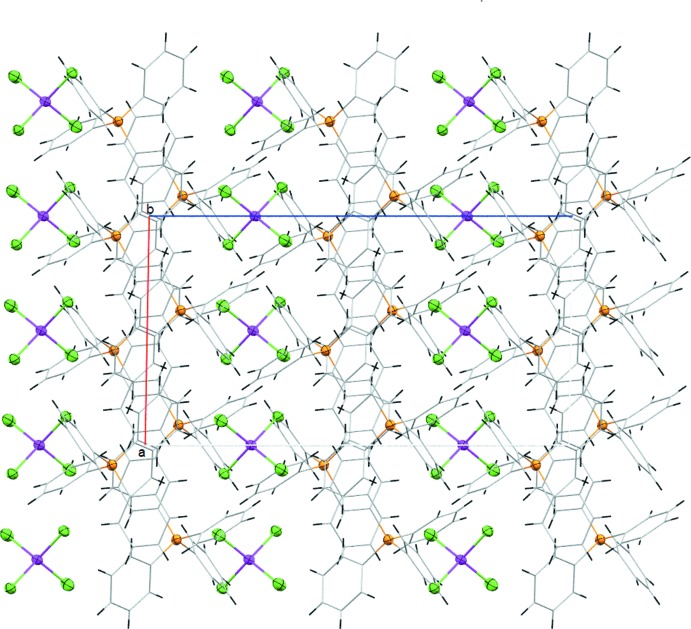
Part of the layered packing in the crystal viewed down the b axis.

**Fig. 3.**
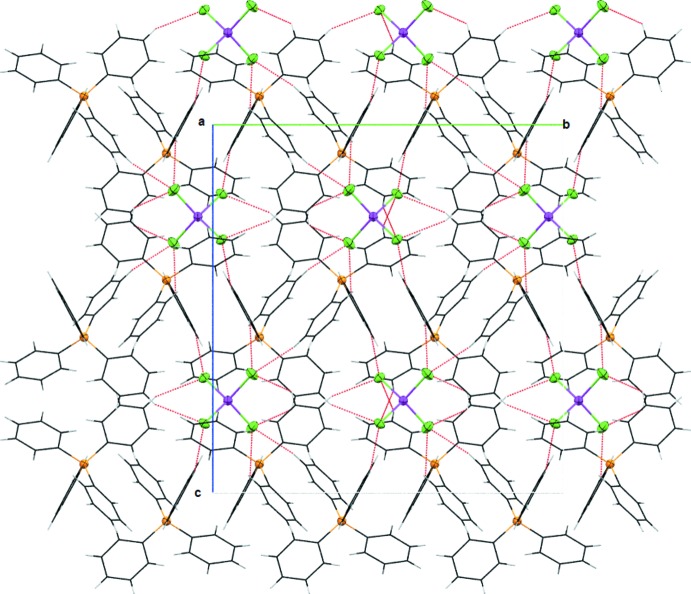
A view along the c axis of the crystal structure of the title compound, showing C—H···Cl hydrogen-bonds.

## supplementary crystallographic information

### 1. Comment

Research on supramolecular compounds has become popular because of their potential applications in areas such as gas storage (Rowsell & Yaghi, 2005), selective absorption (Dong *et al.*, 2007), catalysis (Wu & Lin, 2007), magnetics (Zhao *et al.*, 2003; Neville *et al.*, 2008) and optics (Huang *et al.*, 2007). P-Ligands are important structural motifs in organic syntheses, coordination chemistry and also in various catalytically active compounds, the tetraphenylphosphonium ion have been widely used in supramolecular chemistry and numerous coordination polymers with versatile structures and potential properties have been reported (Zacharie *et al.*, 1985; Schlueter & Geiser, 2007). Thus, we report here the synthesis of title compound [*L*_2_.CoCl_4_], were *L* is tetraphenylphosphonium and its crystal structure.

The asymmetric unit of (I) and atomic numbering are illustrated in Fig. 1. The (I) contains tetraphenylphosphonium cations linked by weak C—H···π supramolecular interactions into dimmers. The Co^II^ ion lies on a twofold axis and has a distorted tetrahedral coordination. The [CoCl_4_]^-2^ anions are linked with the cation via weak C—H···Cl hydrogen bonds (Fig. 2, 3).

The bond lengths for coordination Co^II^ sphere is ranging from 2.2791 (6) to 2.2873 (6) Å for Co—Cl distances (Table 1). The crystal packing in the title structure can be described by altering CoCl_4_ tetrahedral of complex along the *a* axis at b = 1/4 and 3/4 (Fig. 2).

### 2. Experimental

A mixture of CoCl_2_ (2.50 g, 10 mmol), tetraphenylphosphonium chloride hydrate (3.92 g,10 mmol) was dissolved in a 20 ml EtOH/H_2_O(*v*/*v*,1:2). The mixture was then sealed in a 25 ml stainless steel reactor and heated to 433 K for 3 days. Then the reactant mixture was cooled to room temperature at the rate of 5 degrees per hour. Evaporation of the resulting solution for a few days afforded pink crystals of title compound.

### 3. Refinement

The aromatic H atoms were placed at calculated positions with C—H = 0.93, and refined in riding mode with U_iso_(H) = 1.2U_eq_(C).

### Crystal data

**Table:**

(C_24_H_20_P)_2_[CoCl_4_]	*F*(000) = 1812
*M**_r_* = 879.46	*D*_x_ = 1.378 Mg m^−^^3^
Monoclinic, *C*2/*c*	Mo *K*α radiation, λ = 0.71073 Å
Hall symbol: -C 2yc	Cell parameters from 1536 reflections
*a* = 10.9154 (4) Å	θ = 3.2–25.1°
*b* = 19.2514 (6) Å	µ = 0.77 mm^−^^1^
*c* = 20.1826 (7) Å	*T* = 293 K
β = 91.008 (2)°	Prism, pink
*V* = 4240.4 (3) Å^3^	0.2 × 0.1 × 0.08 mm
*Z* = 4	

### Data collection

**Table:**

Bruker APEXII diffractometer	3180 reflections with *I* > 2σ(*I*)
Radiation source: fine-focus sealed tube	*R*_int_ = 0.028
Graphite monochromator	θ_max_ = 25.1°, θ_min_ = 2.0°
φ scans	*h* = −12→12
12226 measured reflections	*k* = −22→22
3748 independent reflections	*l* = −24→22

### Refinement

**Table:**

Refinement on *F*^2^	0 restraints
Least-squares matrix: full	H-atom parameters constrained
*R*[*F*^2^ > 2σ(*F*^2^)] = 0.030	*w* = 1/[σ^2^(*F*_o_^2^) + (0.0401*P*)^2^ + 6.1572*P*] where *P* = (*F*_o_^2^ + 2*F*_c_^2^)/3
*wR*(*F*^2^) = 0.084	(Δ/σ)_max_ = 0.001
*S* = 1.04	Δρ_max_ = 1.51 e Å^−^^3^
3748 reflections	Δρ_min_ = −0.22 e Å^−^^3^
249 parameters	

### Special details

**Table:**

Geometry. Bond distances, angles *etc*. have been calculated using the rounded fractional coordinates. All e.s.d.'s are estimated from the variances of the (full) variance-covariance matrix. The cell e.s.d.'s are taken into account in the estimation of distances, angles and torsion angles
Refinement. Refinement of *F*^2^ against ALL reflections. The weighted *R*-factor *wR* and goodness of fit *S* are based on *F*^2^, conventional *R*-factors *R* are based on *F*, with *F* set to zero for negative *F*^2^. The threshold expression of *F*^2^ > σ(*F*^2^) is used only for calculating *R*-factors(gt) *etc*. and is not relevant to the choice of reflections for refinement. *R*-factors based on *F*^2^ are statistically about twice as large as those based on *F*, and *R*- factors based on ALL data will be even larger.

### Fractional atomic coordinates and isotropic or equivalent isotropic displacement parameters (Å^2^)

**Table:**

	*x*	*y*	*z*	*U*_iso_*/*U*_eq_	
P1	0.41273 (5)	0.36748 (3)	0.07765 (3)	0.0200 (2)	
C1	0.3837 (2)	0.29974 (11)	0.13639 (11)	0.0233 (7)	
C2	0.3155 (2)	0.31597 (12)	0.19182 (11)	0.0308 (7)	
C3	0.2894 (3)	0.26481 (13)	0.23755 (13)	0.0376 (8)	
C4	0.3336 (2)	0.19823 (12)	0.22924 (12)	0.0355 (8)	
C5	0.4026 (2)	0.18228 (12)	0.17495 (12)	0.0335 (8)	
C6	0.4272 (2)	0.23227 (11)	0.12800 (12)	0.0290 (7)	
C7	0.5226 (2)	0.33873 (11)	0.01785 (11)	0.0238 (7)	
C8	0.6408 (2)	0.36542 (13)	0.01635 (12)	0.0291 (7)	
C9	0.7214 (2)	0.34138 (14)	−0.03052 (13)	0.0375 (8)	
C10	0.6855 (2)	0.29065 (13)	−0.07487 (12)	0.0370 (8)	
C11	0.5686 (3)	0.26452 (12)	−0.07432 (12)	0.0363 (8)	
C12	0.4866 (2)	0.28862 (12)	−0.02847 (12)	0.0314 (7)	
C13	0.27533 (19)	0.39129 (10)	0.03328 (10)	0.0199 (6)	
C14	0.2858 (2)	0.42599 (12)	−0.02717 (11)	0.0272 (7)	
C15	0.1824 (2)	0.44748 (12)	−0.06129 (11)	0.0297 (7)	
C16	0.0676 (2)	0.43485 (12)	−0.03560 (11)	0.0269 (7)	
C17	0.0570 (2)	0.40171 (12)	0.02446 (11)	0.0271 (7)	
C18	0.1604 (2)	0.38005 (11)	0.05921 (11)	0.0232 (7)	
C19	0.47179 (19)	0.44088 (11)	0.12202 (10)	0.0205 (6)	
C20	0.4177 (2)	0.50598 (11)	0.11488 (11)	0.0226 (6)	
C21	0.4699 (2)	0.56215 (12)	0.14713 (11)	0.0289 (7)	
C22	0.5746 (2)	0.55408 (13)	0.18543 (11)	0.0304 (7)	
C23	0.6265 (2)	0.48956 (14)	0.19401 (11)	0.0315 (8)	
C24	0.5750 (2)	0.43278 (13)	0.16272 (11)	0.0280 (7)	
Co1	0.00000	0.45721 (2)	0.25000	0.0202 (1)	
Cl1	0.10790 (5)	0.38813 (3)	0.32242 (3)	0.0338 (2)	
Cl2	0.12506 (5)	0.52482 (3)	0.18779 (3)	0.0267 (2)	
H2	0.28760	0.36110	0.19810	0.0370*	
H3	0.24190	0.27530	0.27400	0.0450*	
H4	0.31670	0.16410	0.26040	0.0430*	
H5	0.43290	0.13750	0.16980	0.0400*	
H6	0.47250	0.22100	0.09090	0.0350*	
H8	0.66550	0.39920	0.04670	0.0350*	
H9	0.80030	0.35960	−0.03210	0.0450*	
H10	0.74100	0.27400	−0.10550	0.0440*	
H11	0.54480	0.23060	−0.10480	0.0440*	
H12	0.40700	0.27140	−0.02840	0.0380*	
H14	0.36280	0.43460	−0.04440	0.0330*	
H15	0.18950	0.47050	−0.10160	0.0360*	
H16	−0.00230	0.44880	−0.05900	0.0320*	
H17	−0.02010	0.39380	0.04180	0.0330*	
H18	0.15280	0.35800	0.09990	0.0280*	
H20	0.34740	0.51150	0.08870	0.0270*	
H21	0.43410	0.60580	0.14300	0.0350*	
H22	0.61050	0.59260	0.20570	0.0360*	
H23	0.69600	0.48430	0.22080	0.0380*	
H24	0.60930	0.38900	0.16880	0.0340*	

### Atomic displacement parameters (Å^2^)

**Table:**

	*U*^11^	*U*^22^	*U*^33^	*U*^12^	*U*^13^	*U*^23^
P1	0.0206 (3)	0.0189 (3)	0.0205 (3)	0.0001 (2)	0.0001 (2)	−0.0013 (2)
C1	0.0266 (12)	0.0210 (11)	0.0222 (11)	−0.0004 (9)	−0.0037 (9)	0.0011 (9)
C2	0.0441 (14)	0.0228 (12)	0.0255 (12)	0.0057 (11)	0.0025 (11)	0.0007 (9)
C3	0.0528 (16)	0.0346 (14)	0.0256 (13)	0.0020 (12)	0.0065 (12)	0.0037 (11)
C4	0.0484 (15)	0.0275 (13)	0.0303 (13)	−0.0040 (11)	−0.0064 (12)	0.0096 (10)
C5	0.0454 (15)	0.0187 (11)	0.0361 (14)	0.0038 (11)	−0.0070 (12)	0.0010 (10)
C6	0.0324 (13)	0.0255 (12)	0.0291 (12)	0.0037 (10)	−0.0013 (10)	−0.0025 (10)
C7	0.0272 (12)	0.0207 (11)	0.0235 (11)	0.0042 (9)	0.0018 (9)	0.0013 (9)
C8	0.0240 (12)	0.0355 (13)	0.0279 (12)	0.0022 (10)	−0.0009 (10)	0.0005 (10)
C9	0.0236 (12)	0.0548 (16)	0.0343 (14)	0.0069 (12)	0.0030 (11)	0.0031 (12)
C10	0.0404 (15)	0.0422 (15)	0.0286 (13)	0.0170 (12)	0.0101 (11)	0.0036 (11)
C11	0.0554 (17)	0.0254 (12)	0.0284 (13)	0.0060 (12)	0.0080 (12)	−0.0034 (10)
C12	0.0372 (13)	0.0252 (12)	0.0319 (13)	−0.0035 (10)	0.0056 (11)	−0.0036 (10)
C13	0.0212 (11)	0.0186 (10)	0.0199 (11)	−0.0011 (9)	−0.0018 (9)	−0.0027 (8)
C14	0.0239 (12)	0.0340 (13)	0.0237 (12)	−0.0033 (10)	0.0031 (9)	0.0040 (10)
C15	0.0342 (13)	0.0345 (13)	0.0203 (11)	−0.0004 (11)	0.0002 (10)	0.0061 (10)
C16	0.0260 (12)	0.0286 (12)	0.0258 (12)	0.0038 (10)	−0.0048 (10)	−0.0033 (10)
C17	0.0216 (11)	0.0317 (12)	0.0282 (12)	−0.0009 (10)	0.0035 (9)	−0.0010 (10)
C18	0.0256 (12)	0.0248 (11)	0.0192 (11)	−0.0028 (9)	0.0024 (9)	0.0018 (9)
C19	0.0200 (11)	0.0219 (11)	0.0198 (11)	−0.0015 (9)	0.0036 (9)	−0.0025 (9)
C20	0.0238 (11)	0.0233 (11)	0.0208 (11)	−0.0014 (9)	0.0035 (9)	0.0007 (9)
C21	0.0395 (14)	0.0207 (11)	0.0268 (12)	−0.0033 (10)	0.0079 (11)	−0.0002 (9)
C22	0.0354 (13)	0.0335 (13)	0.0225 (12)	−0.0165 (11)	0.0072 (10)	−0.0080 (10)
C23	0.0228 (12)	0.0483 (15)	0.0235 (12)	−0.0042 (11)	−0.0001 (10)	−0.0070 (11)
C24	0.0253 (12)	0.0324 (13)	0.0262 (12)	0.0054 (10)	−0.0016 (10)	−0.0036 (10)
Co1	0.0188 (2)	0.0189 (2)	0.0231 (2)	0.0000	0.0029 (2)	0.0000
Cl1	0.0315 (3)	0.0335 (3)	0.0367 (3)	0.0084 (3)	0.0074 (3)	0.0151 (3)
Cl2	0.0234 (3)	0.0296 (3)	0.0270 (3)	−0.0035 (2)	0.0015 (2)	0.0071 (2)

### Geometric parameters (Å, º)

**Table:**

Co1—Cl2	2.2791 (6)	C19—C24	1.391 (3)
Co1—Cl1^i^	2.2873 (6)	C19—C20	1.392 (3)
Co1—Cl1	2.2873 (6)	C20—C21	1.380 (3)
Co1—Cl2^i^	2.2791 (6)	C21—C22	1.377 (3)
P1—C1	1.794 (2)	C22—C23	1.375 (4)
P1—C7	1.803 (2)	C23—C24	1.377 (3)
P1—C13	1.793 (2)	C2—H2	0.9301
P1—C19	1.787 (2)	C3—H3	0.9296
C1—C6	1.394 (3)	C4—H4	0.9302
C1—C2	1.390 (3)	C5—H5	0.9298
C2—C3	1.383 (3)	C6—H6	0.9302
C3—C4	1.381 (3)	C8—H8	0.9301
C4—C5	1.375 (3)	C9—H9	0.9310
C5—C6	1.380 (3)	C10—H10	0.9301
C7—C8	1.390 (3)	C11—H11	0.9310
C7—C12	1.395 (3)	C12—H12	0.9300
C8—C9	1.383 (3)	C14—H14	0.9301
C9—C10	1.377 (4)	C15—H15	0.9309
C10—C11	1.372 (4)	C16—H16	0.9299
C11—C12	1.379 (4)	C17—H17	0.9297
C13—C14	1.397 (3)	C18—H18	0.9295
C13—C18	1.385 (3)	C20—H20	0.9301
C14—C15	1.375 (3)	C21—H21	0.9299
C15—C16	1.386 (3)	C22—H22	0.9302
C16—C17	1.376 (3)	C23—H23	0.9293
C17—C18	1.383 (3)	C24—H24	0.9295
			
Cl1—Co1—Cl2	112.17 (2)	C22—C23—C24	119.7 (2)
Cl1—Co1—Cl1^i^	108.90 (3)	C19—C24—C23	120.1 (2)
Cl1—Co1—Cl2^i^	106.66 (2)	C3—C2—H2	120.17
Cl1^i^—Co1—Cl2	106.66 (2)	C1—C2—H2	120.06
Cl2—Co1—Cl2^i^	110.35 (3)	C2—C3—H3	119.85
Cl1^i^—Co1—Cl2^i^	112.17 (2)	C4—C3—H3	119.91
C13—P1—C19	109.84 (10)	C3—C4—H4	119.98
C1—P1—C19	108.05 (10)	C5—C4—H4	119.92
C1—P1—C7	110.27 (10)	C6—C5—H5	119.72
C1—P1—C13	111.10 (10)	C4—C5—H5	119.80
C7—P1—C13	107.73 (10)	C1—C6—H6	120.13
C7—P1—C19	109.86 (10)	C5—C6—H6	120.17
P1—C1—C6	122.16 (17)	C7—C8—H8	120.18
P1—C1—C2	118.14 (17)	C9—C8—H8	120.26
C2—C1—C6	119.7 (2)	C10—C9—H9	119.85
C1—C2—C3	119.8 (2)	C8—C9—H9	119.83
C2—C3—C4	120.2 (2)	C9—C10—H10	119.68
C3—C4—C5	120.1 (2)	C11—C10—H10	119.72
C4—C5—C6	120.5 (2)	C12—C11—H11	120.17
C1—C6—C5	119.7 (2)	C10—C11—H11	120.04
P1—C7—C8	121.99 (17)	C7—C12—H12	119.84
P1—C7—C12	118.55 (17)	C11—C12—H12	119.90
C8—C7—C12	119.5 (2)	C13—C14—H14	120.00
C7—C8—C9	119.6 (2)	C15—C14—H14	119.88
C8—C9—C10	120.3 (2)	C16—C15—H15	120.01
C9—C10—C11	120.6 (2)	C14—C15—H15	120.04
C10—C11—C12	119.8 (2)	C15—C16—H16	119.90
C7—C12—C11	120.3 (2)	C17—C16—H16	120.01
C14—C13—C18	119.55 (19)	C16—C17—H17	119.87
P1—C13—C18	121.79 (16)	C18—C17—H17	119.73
P1—C13—C14	118.54 (16)	C17—C18—H18	120.08
C13—C14—C15	120.1 (2)	C13—C18—H18	120.05
C14—C15—C16	120.0 (2)	C19—C20—H20	120.40
C15—C16—C17	120.1 (2)	C21—C20—H20	120.54
C16—C17—C18	120.4 (2)	C20—C21—H21	119.77
C13—C18—C17	119.9 (2)	C22—C21—H21	119.71
P1—C19—C24	119.24 (17)	C23—C22—H22	119.70
P1—C19—C20	120.76 (16)	C21—C22—H22	119.73
C20—C19—C24	120.0 (2)	C22—C23—H23	120.14
C19—C20—C21	119.1 (2)	C24—C23—H23	120.18
C20—C21—C22	120.5 (2)	C23—C24—H24	119.93
C21—C22—C23	120.6 (2)	C19—C24—H24	119.98

Symmetry code: (i) −*x*, *y*, −*z*+1/2.

### Hydrogen-bond geometry (Å, º)

Cg2 and Cg4 are the centroids of the C19–C24 and C7-C12 benzene rings, respectively.

**Table:**

*D*—H···*A*	*D*—H	H···*A*	*D*···*A*	*D*—H···*A*
C3—H3···Cl1	0.93	2.80	3.552 (3)	138
C11—H11···Cl1^ii^	0.93	2.81	3.633 (2)	148
C23—H23···Cl2^iii^	0.93	2.77	3.644 (2)	156
C14—H14···*Cg*4^iv^	0.93	2.88	3.650 (2)	141
C21—H21···*Cg*2^iv^	0.93	2.79	3.446 (2)	129

Symmetry codes: (ii) *x*+1/2, −*y*+1/2, *z*−1/2; (iii) −*x*+1, *y*, −*z*+1/2; (iv) −*x*+1, −*y*+1, −*z*.

## References

[bb1] Bruker (2006). *APEX2* and *SAINT* Bruker AXS Inc. Madison, Wisconsin, USA.

[bb2] Burla, M. C., Camalli, M., Carrozzini, B., Cascarano, G. L., Giacovazzo, C., Polidori, G. & Spagna, R. (2003). *J. Appl. Cryst.* **36**, 1103.

[bb3] Dong, Y.-B., Zhang, Q., Liu, L.-L., Ma, J.-P., Tang, B. & Huang, R.-Q. (2007). *J. Am. Chem. Soc.* **129**, 1514–1515. 10.1021/ja067384z 17249672

[bb4] Farrugia, L. J. (2012). *J. Appl. Cryst.* **45**, 849–854.

[bb5] Huang, Y.-L., Huang, M.-Y., Chan, T.-H., Chang, B. C. & Lii, K. L. (2007). *Chem. Mater.* **19**, 3232–3237.

[bb6] Macrae, C. F., Edgington, P. R., McCabe, P., Pidcock, E., Shields, G. P., Taylor, R., Towler, M. & van de Streek, J. (2006). *J. Appl. Cryst.* **39**, 453–457.

[bb7] Neville, S. M., Halder, G. J., Chapman, K. W., Duriska, M. B., Southon, P. D., Cashion, J. D., Letard, J. F., Moubaraki, B., Murray, K. S. & Kepert, C. J. (2008). *J. Am. Chem. Soc.* **130**, 2869–2876. 10.1021/ja077958f 18254628

[bb8] Persistence of Vision Team (2004). *POV-RAY* Persistence of Vision Raytracer Pty Ltd, Victoria, Australia. URL: http://www.povray.org/.

[bb9] Rowsell, J. L. C. & Yaghi, O. M. (2005). *Angew. Chem. Int. Ed.* **44**, 4670–4679. 10.1002/anie.200462786 16028207

[bb10] Schlueter, J. A. & Geiser, U. (2007). *Acta Cryst.* C**63**, m235–m237. 10.1107/S0108270107015387 17551176

[bb11] Sheldrick, G. M. (2008). *Acta Cryst.* A**64**, 112–122. 10.1107/S0108767307043930 18156677

[bb12] Wu, C.-D. & Lin, W.-B. (2007). *Angew. Chem. Int. Ed.* **46**, 1075–1078.

[bb13] Zacharie, B., Wuest, J. D., Olivier, M. J. & Beauchamp, A. L. (1985). *Acta Cryst.* C**41**, 369–371.

[bb14] Zhao, B., Cheng, P., Dai, Y., Cheng, C., Liao, D.-Z., Yan, S.-P., Jiang, Z.-H. & Wang, G.-L. (2003). *Angew. Chem. Int. Ed.* **42**, 934–936. 10.1002/anie.200390248 12596182

